# Metagenomic 16S rDNA amplicon data of microbial diversity and its predicted metabolic functions in the Southern Ocean (Antarctic)

**DOI:** 10.1016/j.dib.2019.104876

**Published:** 2019-11-23

**Authors:** Balamurugan Sadaiappan, Sivakumar Kannan, Sivasankar Palaniappan, Radhakrishnan Manikkam, Balagurunathan Ramasamy, Anilkumar N., Mahendran Subramanian

**Affiliations:** a Plankton Ecology Laboratory, CSIR National Institute of Oceanography, Goa, India; b Centre of Advanced Study in Marine Biology, Annamalai University, Tamil Nadu, India; c Department of Microbiology, Periyar University, Salem, India; d Centre for Drug Discovery and Development, Sathyabama Institute of Science and Technology, Chennai, India; e National Centre for Antarctic and Ocean Research, Goa, India; f Department of Bioengineering and Department of Computing, Imperial College London, United Kingdom

**Keywords:** Microbial diversity, Southern ocean, Metabolic functions, Proteobacteria

## Abstract

Antarctica holds about 70% of all the freshwater on the planet in the form of ice. The seawater, it chills, affect the currents and temperature everywhere. Global warming risks the melting of the icecaps as it has already increased the ocean temperature by 1 °C to the West Antarctic peninsula since 1955. A better understanding of the microbial community in this extreme environment of utmost importance is of interest to the scientific community. Herein, we document our metagenomics analysis of the microbial diversity and abundance in the Southern Ocean [Lat 55″ 33′ 396 S; Lon 55″ 31′ 448 E] using Next Generation Sequencing (NGS), QIIME 1.9.1, Silvangs and a naïve Bayesian classifier. Such metagenomics data hold the potential to aid predictive analysis, which is critical to our understanding of the dynamics of the microbial communities and their role in the Southern Ocean at present and in the future.

**Table:** Specifications Table

Subject	Microbial Bioinformatics
Specific subject area	Metagenomic study on the microbial community in the Southern Ocean (Antarctic).
Type of data	Figures, Table and 16S rDNA illumina sequence
How data were acquired	The surface seawater sample was collected using a Niskin bottle fitted in the CTD and filtered using 47mm dia 0.2 μm polycarbonate filter paper. The eDNA was isolated using DNasy PowerWater Kit (Qiagen), and sequenced was carried in illumine Miseq.
Data format	Data in fastq format were deposited in the NCBI SRA https://www.ncbi.nlm.nih.gov/sra.
Description of data collection	The eDNA was isolated from the 47mm dia 0.2 μm diameter polycarbonate filter paper, and 16S rDNA region was amplified using V3–V4 primer, and paired-end sequencing was carried out using illumine Miseq platform.
Data source location	The water samples were collected during the Southern Ocean (Antarctica) expedition SOE 8 (9th January to 24th February, 2015) at the coordinates of 55″ 33′ 396 S; 55″ 31′ 448 E, using Niskin sampler on board and the temperature, salinity and oxygen were obtained using onboard Seabird CTD.
Data accessibility	The sequence was submitted to the public repository and the accession number is SRR6129935. https://www.ncbi.nlm.nih.gov/sra/?term=SRR6129935

**Table:** 

**Value of the data** ➢ The metagenomics data documented here provide insights into the microbial diversity and abundance in the Southern Ocean [Lat 55″ 33′ 396 S; Lon 55″ 31′ 448 E]. ➢ The data also shines a light on the microbial communities' metabolic functions in the Southern Ocean. ➢ The data can be used to prepare 16S rDNA primers for next-generation sequencing. ➢ The metagenomics data hold the potential to aid predictive analysis, which is critical to our understanding of the dynamics of the microbial communities and their role in the Southern Ocean at present and in the future.

## 1 Data

**Fig. 1:**
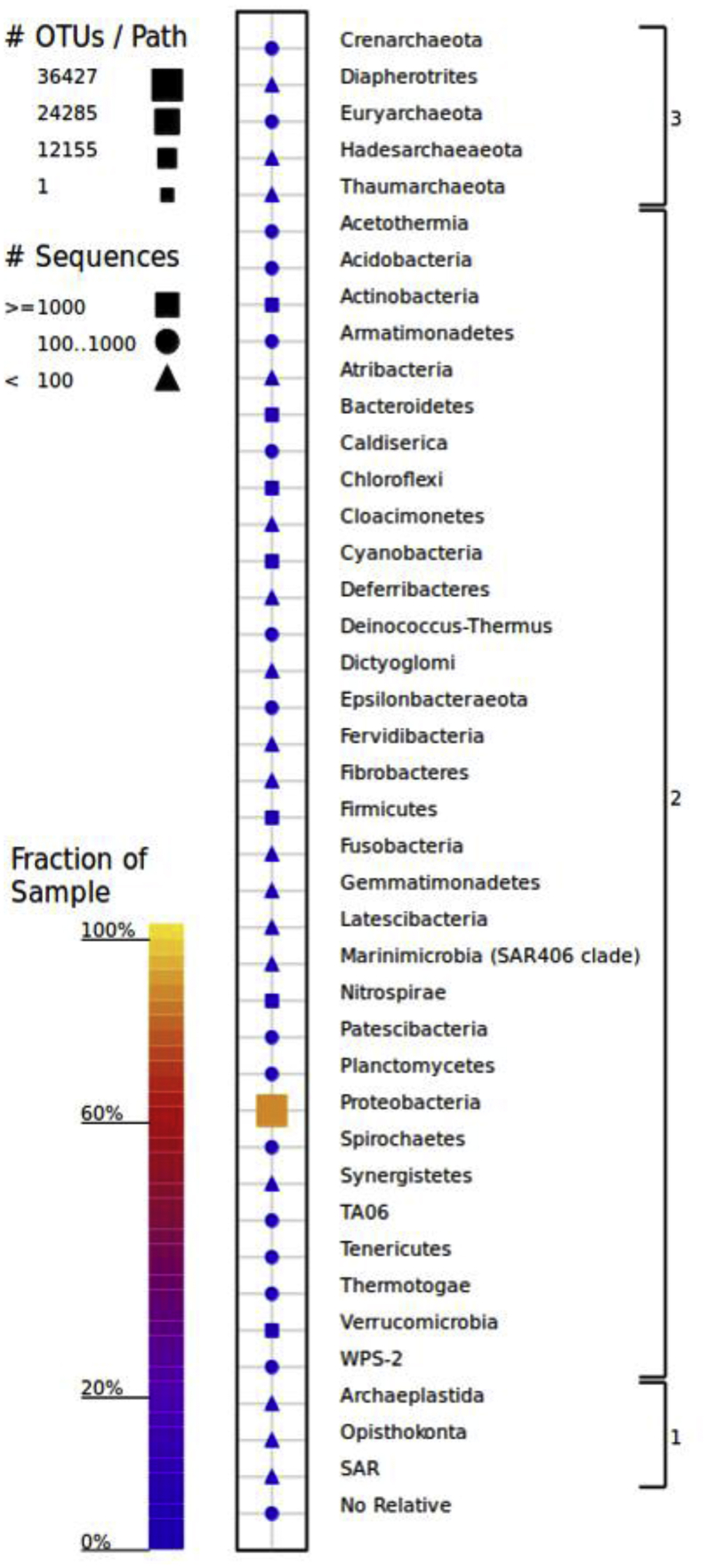
Occurrence and abundance of different Phyla in the Southern Ocean (Antarctica).

The illumina Miseq sequencer produce 724,150 paired end reads, after quality filter and contigs assembly with average read length of 250bp we obtained 362,075 reads. The Qiime191 analysis showed that the reads represented two microbial Kingdoms (Bacteria and Archaea). Overall, the reads represented 38 phyla, 86 classes, and 412 genera. The bacterial diversity was found to be higher, with 96.8% of the total reads, followed by Archaea with only 1.7%. Among the diversity the bacterial diversity varied widely and included 33 phyla viz. Acidobacteria, Actinobacteria, Bacteroidetes, Chloroflexi, Cyanobacteria, Deinococcus-Thermus, Firmicutes, Nitrospirae, Planctomycetes, Proteobacteria, Verrucomycetes and 1.5% of the total reads were unclassified (Fig. 1).

**Fig. 2:**
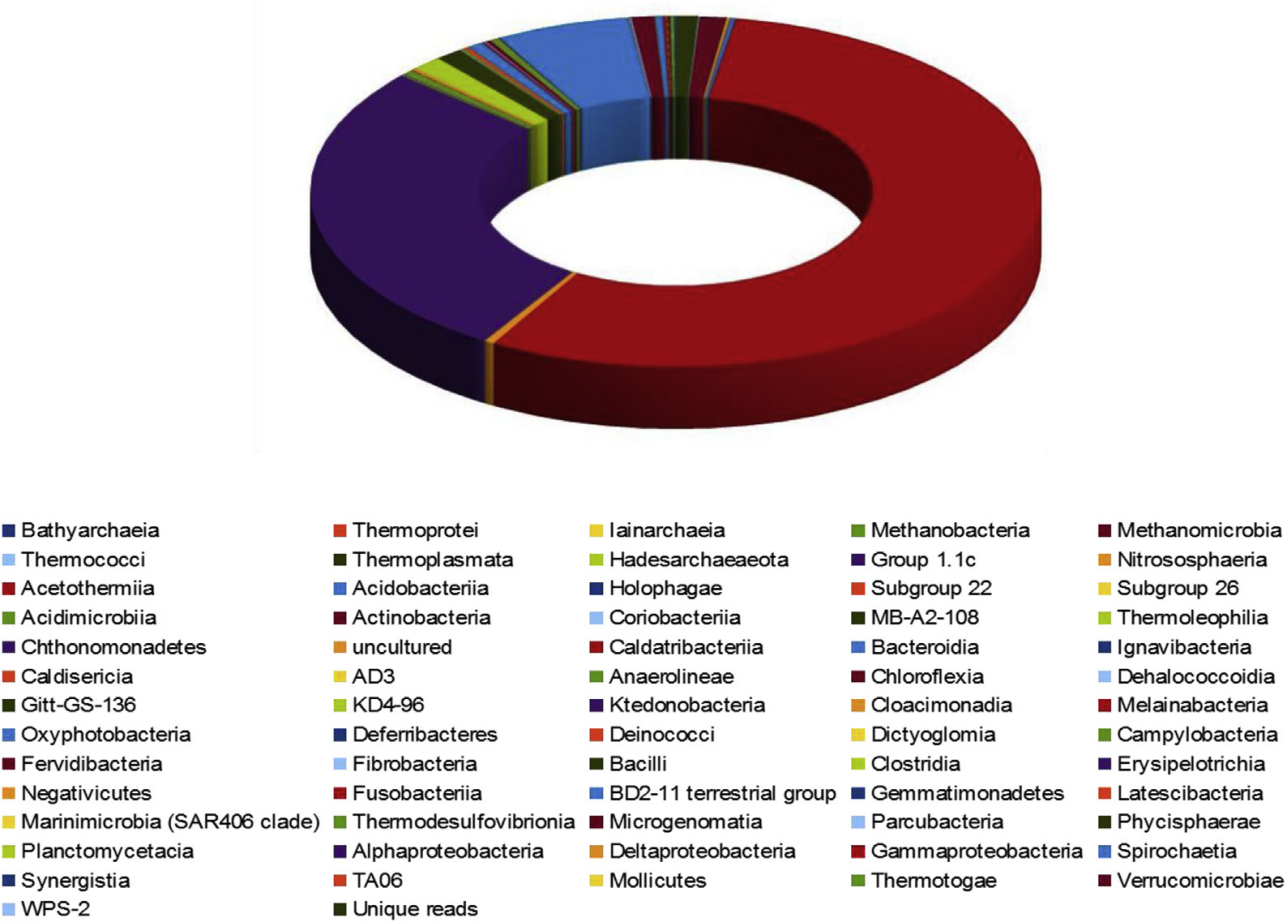
Relative abundances of bacterial (96.8%) archaeal (1.7%) classes and unique reads (1.5%) in water sample of Southern Ocean.

Within the bacterial fractions, the Phylum Proteobacteria was dominant, with 87% of the total reads consisted of 3 classes’ viz. Gammaproteobacteria (58%), Alphaproteobacteria (29%), and Deltaproteobacteria (0.4%) (Fig. 2). Followed by phylum Bacteroidetes (4.2%), Firmicutes (1.70%). Archaeal diversity, consisted of 5 Phyla such as Crenarchaeota, Diapherotrites, Euryarchaeaota, Hadesarchaeaota and Thaumarchaeota.

## 2 Experimental design, material and methods

### 2.1 Sample collection

The surface seawater samples were collected during the Southern Ocean (Antarctica) expedition SOE 8 (Nov 2016) at the coordinates of 55″ 33′ 396 S; 55″ 31′ 448 E, using Niskin water sampler on board and the temperature, salinity and oxygen were obtained using on board Seabird CTD (Table 1). The water samples were stored in −80 °C and transported to the CAS in Marine Biology laboratory for further studies.

**Table 1:** Physico-chemical parameters.

Parameter (units)	Values
O2 (μM)	347.9563
pH	7.85
NO3 (μM)	26.54
NO_2_ (μM)	0.44
PO_4_ (μM)	0.41
SiO_4_ (μM)	22.34

### 2.2 DNA isolation

Water samples were filtered through 47mm dia 0.2 μm polycarbonate filter paper, and the eDNA was isolated using DNeasy PowerWater Kit (Qiagen). Briefly, the filter papers were homogenised with 5ml of lysis buffer and incubated for 90 min, and the mixture was centrifuged at 4000g for 5 mins at 4 °C. The supernatant was collected in a separate fresh tube, and the DNA was precipitated by adding ice-cooled 0.7 vol isopropanol. The isolated DNA was pooled together, and the quality was checked in agarose and NanoDrop 2000/2000c (Thermos Scientifics). The extracted DNA was sent to the Xcelris Labs Ltd, Ahmedabad, India, for amplicon sequencing.

### 2.3 DNA sequence

The amplicon libraries were prepared using the Nextera XT Index Kit (Illumina inc.) as per the 16S Metagenomics Sequencing Library preparation protocol (Part # 15044223 Rev. B). The V3–V4 hypervariable region of 16S rDNA gene of bacteria and archaea primer was designed in Xcelris NGS Bioinformatics Lab [1]. These primers were synthesised within the Xcelris PrimeX facility. The amplicons with the Illumina adaptors were amplified, using i5 and i7 primers that add multiplexing index sequences as well as standard adapters required for cluster generation (P5 and P7) as per the standard Illumina protocol. The amplicon libraries were purified by 1X AmpureXP beads and checked on Agilent High Sensitivity (HS) chip on Bioanalyzer 2100 and quantified on fluorimeter by Qubit dsDNA HS Assay kit (Life Technologies). The library was loaded onto the Illumina Platform at an appropriate concentration (10–20pM) for cluster generation and sequencing. Paired-End sequencing allows the template fragments to be sequenced in both the forward and reverse directions.

### 2.4 Sequence analysis

**Fig. 3:**
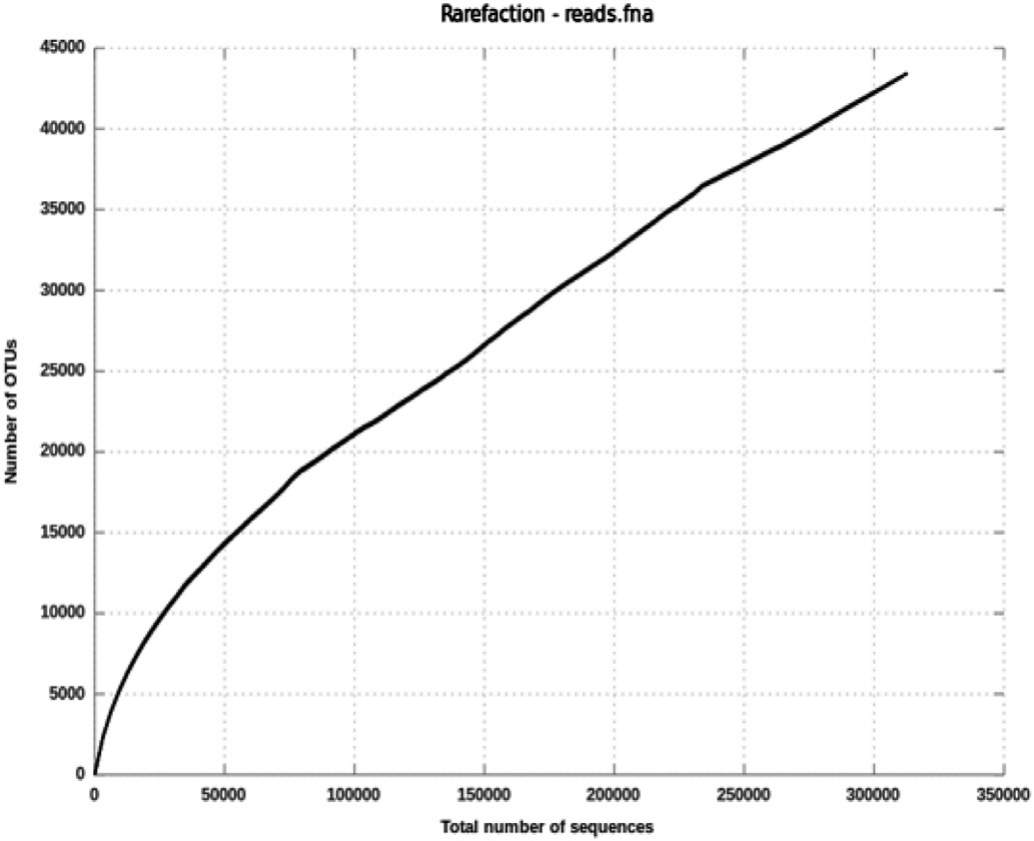
Rarefaction curve showing the inadequate sampling.

**Fig. 4:**
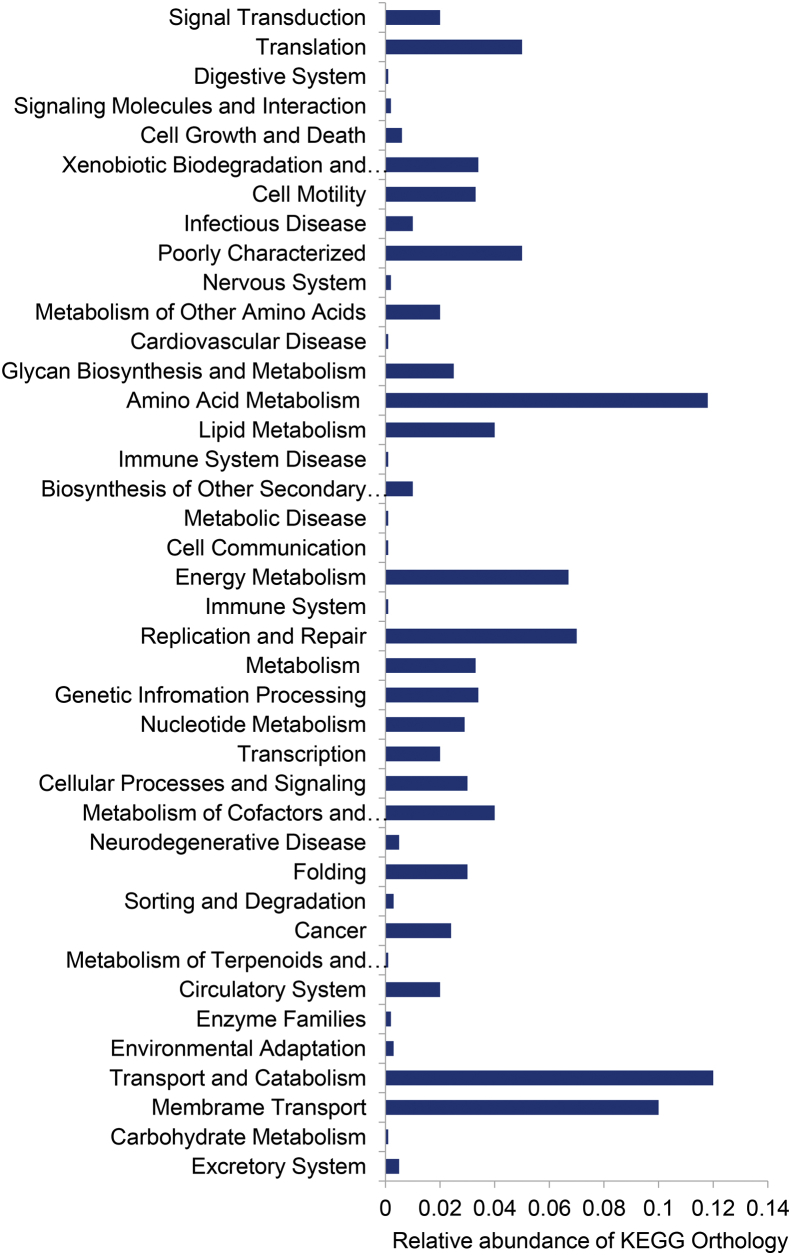
The predicted metabolic functions of the Southern Ocean.

The sequences were analysed in QIIME 1.9.1 [2] and only the sequence with minimum 150bp length was used for further analysis. The bacterial and archaeal 16S rDNA sequences were clustered at 97% using both the open and closed-reference OTU picking a strategy and the OTUs were classified using Greengene 13_8 16S reference database [3]. The taxonomy assignment was made to each Operational Taxonomic Unit (OTU) using the RDP classifier [4] and Silvangs [5]. The sequence coverage was evaluated by rarefaction analysis (Fig. 3), and the species richness (H = 5.7) and diversity index values (Chao1 = 1439) were calculated. The microbial metabolic pathways were estimated based on the 16S rDNA gene data using Parallel-Meta3 software [6] were a core-housekeeping function such as amino acid metabolism and lipid metabolism was dominant followed by the biosynthesis of other secondary metabolites, xenobiotics, degradation and metabolism and nucleotide metabolism (Fig. 4). NSTI value was determined in the present study, and it was 0.10085 indicating that the predicted functions of the microbial taxa are very close to the microbial reference genome database, and it shows the accuracy of the prediction.
